# Nephritis Following the Use of Certain of the Balsams

**Published:** 1907-04-13

**Authors:** John Allan


### The General Practitioners' Column.

### NEPHRITIS FOLLOWING THE USE OF CERTAIN OF THE BALSAMS.

By JOHN ALLAN, M.B., Ch.B.Edin.

Attention should, I think, be drawn to a fact which is not generally known, or at any rate not so widely known as it should be—namely, the production of nephritis by the use of certain balsams in the treatment of scabies. There have been recorded in German periodicals a few cases in which the serious condition of the patient was not recognised until the symptoms of acute nephritis had appeared. Having these cases in mind, I decided to watch carefully the effect of storax in some cases of scabies which came under treatment during the course of last year. All the cases were very chronic, and the disease had been going on for some weeks before they came under notice.

W. C——, a little boy two years of age, first came under observation on September 1, 1906, and was found to be suffering from scabies, which was of some weeks' duration. The urine was examined and was found to be free from albumen. The following ointment was prescribed to be rubbed into the affected parts twice daily:—

| | |
|---|---|
| Styracis ... | ℥j. |
| Ol. olivæ ... | ℥ss. |
| Ceræ flavæ ... | ʒij. |

On the following Wednesday (September 5) the child was admitted to the Children's Hospital, Brighton, and, on examination, the urine was found to contain a considerable quantity of albumen. Sulphur ointment was substituted for the above, and three days later white precipitate ointment was employed. The urine did not contain any blood, and on microscopic examination no blood corpuscles nor tube casts could be detected. The albumen got gradually less, and by September 13 had disappeared from the urine. The disease had improved greatly, and was, indeed, cured with the exception of one small area on the left forearm and one small area on the dorsum of the left foot, where the disease appeared to be still active. To these areas the unguentum styracis was applied night and morning. On September 15 albumen in considerable quantity was again found in the urine. The sulphur ointment was again used, and this finally succeeded in stamping out the disease.

A second case was that of A. T——, a boy 4½ years of age, who came under treatment for extreme wasting. He also had scabies which had been going on for a considerable time. Under treatment with sulphur ointment and white precipitate ointment considerable improvement took place, but the disease was not cured. On September 13, 1906, as no albumen was found in the urine, the unguentum styracis was applied. On September 16 albumen appeared in the urine, and so the storax was discontinued and unguentum sulphuris again applied. This had the desired effect, and he left hospital five days later free from the disease.

Three other cases which were observed reacted in the same way.

If the storax had been pushed in these cases it is very probable that acute nephritis would have supervened. The fact that the albumen appeared in the urine after the storax ointment was applied and disappeared when that was stopped, and the further fact that of five cases which were carefully observed all reacted in the same way, prove that the albuminuria could not be mere coincidence. The cases, too, give a good illustration of the difficulty experienced in curing scabies if the condition is allowed to become chronic.

In testing for the albumen the cold nitric acid test was not relied on, for it is well known that nitric acid, or indeed any acid, precipitates the oleo resins.

I have been unable to find any record of this phenomenon by an English writer. In the *British Journal of Dermatology*, 1904, page 440, there is an abstract of Gassmann's paper (*Mun. Med. Woch.*, July 26, 1904). A man, age twenty-six, affected with scabies, had half the body treated with balsam of Peru vaseline for two nights. Acute nephritis supervened. Three per cent. of albumen was found, and there were tube casts and blood in the urine. In the *British Journal of Dermatology*, 1906, page 413, there is an abstract of a paper by Richarty (*Mun. Med. Woch.*, May 8, 1906). This refers to a patient sixteen years of age, treated for scabies with 10 per cent. balsam of Peru. There were three applications. Acute nephritis was produced, and death occurred in fourteen days.

These cases should cause one to exercise great care and judgment in using storax and balsam of Peru in such cases, but I do not think that their use is absolutely vetoed by these records. The production of albuminuria by them is no doubt alarming, but it is transient if they are discontinued as soon as the albumen appears. Sulphur ointment succeeds in curing the disease in the vast majority of cases, but in chronic scabies it appears to be occasionally insufficient to bring about a cure. In these obstinate cases storax or balsam of Peru, carefully applied, seems to afford a means of curing the disease.

I think it proper that the attention of the profession should be directed to the danger attending the use by inunction of storax and balsam of Peru, and I would urge that the urine of cases treated by such means should be frequently examined for albumen, and as soon as any albumen is detected their application should be at once discontinued.

I am indebted to Dr. A. W. Williams for permission to make use of the above cases.

